# Functional MRI Examination of Visual Pathways in Patients with Unilateral Optic Neuritis

**DOI:** 10.1155/2012/265306

**Published:** 2012-07-17

**Authors:** Giulia Mascioli, Simone Salvolini, Gian Luca Cavola, Mara Fabri, Alfonso Giovannini, Cesare Mariotti, Luca Salvolini, Gabriele Polonara

**Affiliations:** ^1^Sezione Scienze Radiologiche, Dipartimento di Scienze Cliniche Specialistiche ed Odontostomatologiche, Università Politecnica delle Marche, Via Tronto 10/A, 60126 Ancona, Italy; ^2^Sezione Neuroscienze Cliniche, Dipartimento di Medicina Sperimentale e Clinica, Università Politecnica delle Marche, 60121 Ancona, Italy; ^3^Sezione di Neuroscienze e Biologia Cellulare, Dipartimento di Medicina Sperimentale e Clinica, Università Politecnica delle Marche, 60121 Ancona, Italy

## Abstract

The relations between brain areas involved in vision were explored in 8 patients with unilateral acute optic neuritis using functional magnetic resonance imaging (fMRI) and diffusion tensor imaging (DTI). In all patients monocular stimulation of affected and unaffected eye elicited significantly different activation foci in the primary visual cortex (V1), whereas the foci evoked in the middle temporal visual area (area V5) were similar in size and in delay of blood-oxygen-level-dependent response. DTI analysis documented lower white matter anisotropy values and reduced fibre reconstruction in the affected compared with the unaffected optic nerves. The preserved activation of area V5 observed in all our patients is an interesting finding that suggests the notion of a different sensitivity of the optic pathways to inflammatory changes.

## 1. Introduction 

Optic neuritis (ON) is an inflammatory disease of the optic nerves characterized by retrobulbar pain, unilateral vision impairment or loss, visual field defects, and impaired contrast and colour sensitivity [1, 2]. Evolution usually involves spontaneous recovery of vision after resolution of the inflammation and remyelinization, by virtue of cortical and subcortical visual pathway neuronal plasticity, but progression to monocular blindness may take place in a significant number of cases [1, 3–5]. Several studies of functional recovery after brain injury have suggested that changes in local connectivity [6, 7], recruitment of existing pathways, or creation of new pathways [8] may account for recovery of vision in these patients. For instance, the evidence of preserved visual function despite a damaged primary visual cortex (V1) in primates has generated the hypothesis of alternative visual pathways involving the lateral geniculate nucleus (LGN) and the middle temporal area (V5) [9–12]. In this study the responses of the visual cortex to monocular visual stimuli were investigated by functional magnetic resonance imaging (fMRI) to elucidate the connections among the brain areas involved in vision during unilateral vision loss in patients with a damaged optic nerve but a healthy visual cortex. 

## 2. Materials and Methods 

### 2.1. Subjects

fMRI and diffusion tensor imaging (DTI) data were collected in 8 patients with acute ON (mean age 46 years, 5 women; Table 1). In 4 patients ON was the first manifestation of multiple sclerosis and 2 further patients had, respectively, optic nerve damage connected to maxillofacial injury and ischemic optic neuropathy; the remaining two cases were classified as idiopathic, because of negative MRI findings and the absence of any clear cause. 

Exhaustive ophthalmological examination and perimetry testing demonstrated unilateral vision loss or impairment (4 in the left eye and 4 in the right eye). Visual acuity in the affected eye ranged from 20/200 (1.0 LogMAR) to 20/125 (0.8 LogMAR) in all patients.

**Figure 1:**
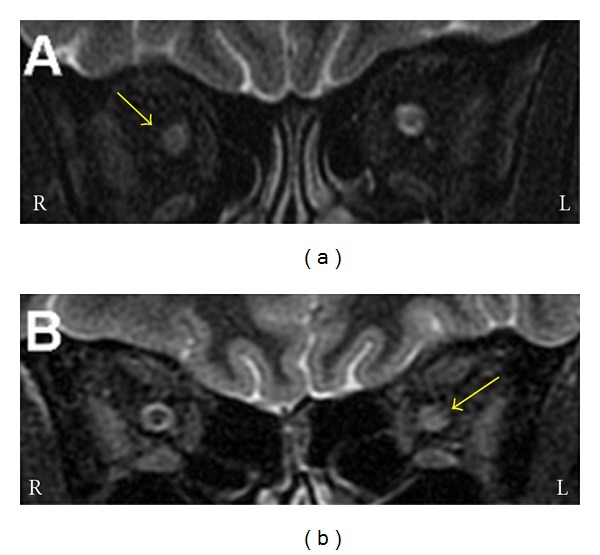
Coronal images of the orbits of two patients with acute right (a) and left (b) optic neuritis obtained with an inversion recovery (STIR) sequence. (a) The anterior part of the right optic nerve is swollen. The perioptic space is less obvious. (b) The posterior portion of the left optic nerve is bright and swollen. Yellow arrows: affected optic nerve. L and R: left and right.

MR coronal images obtained with STIR sequences showed hyperintense areas in the affected optic nerves in all patients (Figure 1). No signal alterations were detected in the visual cortex in either occipital pole. 

### 2.2. Imaging Protocols

Subjects were placed in a 1.5-Tesla (T) scanner (Signa Excite NV/i CV/i, General Electric Medical System, Milwaukee, WI, USA) equipped with 50 mT/m gradients with their head restrained within a circularly polarized head coil. Morphological brain MR images were obtained using sagittal Spin-echo T1-weighted sequences, axial FLAIR and T2-weighted Fast Spin-echo sequences, and coronal T2-weighted sequences. The optic nerve was explored using a coronal STIR sequence (section thickness, 3 mm). 

#### 2.2.1. Functional Imaging

The experimental procedure consisted in the acquisition of a 3D data set (IR Prep Fast SPGR 3-D; TR 15.2 ms, TE 6.9 ms, TI 500 ms, Flip Angle 15°, FOV 29 × 29 cm, slice thickness 1 mm, matrix 288 × 288, 1 Nex, and scan time 8:20 min) and of 10 contiguous 5-mm-thick axial high-resolution anatomical images (T1 FLAIR, 2D, TR 1700 ms, TE 24 ms, Field of View 24 × 24 cm, thickness 5 mm, Matrix 256 × 256, 1 Nex, scan time 2:25 min for 10 images) on which the functional activations were overlaid. fMRI data were then acquired in the same axial planes with a single-shot T2*-weighted gradient-echo EPI sequence (TR 3000 ms, TE 60 ms, Flip Angle 90°, Field of View 24 × 24 cm, Matrix 64 × 64, 1 Nex, scan time 5:12 min) to obtain, during the stimulation cycle, 1000 axial functional images (100/section, 1 image/3 s) from the 10 contiguous 5-mm-thick axial sections selected.

#### 2.2.2. Diffusion Tensor Imaging

Axial diffusion-weighted images with a diffusion sensitising gradient (*b* value = 1000 s/mm^2^) were obtained for diffusion tensor analysis using a single shot spin-echo echo-planar sequence with the following parameters: TR = 6500 msec, TE = 7.4 msec, matrix = 128 × 128, FOV = 24 × 24 cm, slice thickness = 5.0 mm, interslice gap = 1.0 mm, Nex = 2, and scan time 5:51 min. Diffusion was measured along 25 noncollinear directions. 

#### 2.2.3. Visual Stimulation

Visual stimuli-generated using an in-house software were projected in a head-mounted display. A black and white chessboard (amplitude: 6°) was presented to the centre of the visual field (CVF) to an eye at a time (virtual Cartesian distance from viewer's eyes: 75 cm) using a 5 min block-design experimental paradigm alternating twenty 15-s periods of rest and stimulation. Patients were instructed to fix their gaze on a cross in the centre of the display throughout the visual studies. Eye movements were monitored with an internal camera. 

### 2.3. Data Analysis 

#### 2.3.1. Functional Imaging

After each experimental session the images were transferred to a Unix workstation (General Electric Advantage Windows 4.2) and then to a personal computer. Data were analyzed with the BrainVoyager QX software (Brain Innovation, Maastricht, the Netherlands). The first two images of each functional series were discarded to take into account the period of signal intensity variation related to progressive saturation. Data from each subject were preprocessed to remove noise and artefacts. The functional images of each subject were overlaid on the 2D anatomical images and coregistered into their 3D data sets through trilinear interpolation. Data were then transformed into Talairach space [13]. Each scan was adjusted to the Talairach coordinate system to identify the position of activated areas. 

Statistical analysis was applied to the data from each patient using the general linear model (GLM) [14], which aims to predict the variation of a dependent variable (fMRI time course) in terms of linear combination. To account for the haemodynamic delay the predictor time course was convolved with a standard haemodynamic response function. When the signal increase was significantly different from the baseline and correlated temporally with the stimulation pattern, activation was assumed to be evoked by the peripheral stimulus. Activated volumes were considered to consist of clusters of at least 8 voxels (1 voxel = 1 mm^3^). 

#### 2.3.2. Diffusion Tensor Imaging

The images were transferred to the Unix workstation, where DTI data were postprocessed using Functool 3.1.22 (GE Medical Systems). Echo-planar imaging distortion was automatically corrected. Diffusion eigenvectors and eigenvalues, reflecting the main direction of diffusion and associated diffusivity, were calculated from the diffusion tensor. Anisotropy was calculated using orientation-independent fractional anisotropy (FA). The FiberTrak option allows Functool DTI processing to create 2D colour orientation maps, 2D colour eigenvector maps, and 3D tractography maps. The 3D volume viewer permits areas with high FA to be displayed as 3D images. The anisotropy threshold for tracking termination was 0.18. Regions of interest (ROIs) measuring 105 mm^2^ were selected in the occipital visual cortex to visualize the optic radiations. Other ROIs were selected in the retrobulbar segment of the optic nerve and along the retrochiasmatic pathway, to reconstruct the fibres crossing through them (using the origin and destination regions method). All ROIs were defined manually on axial colour-coded maps of the main diffusion directions. 

The mean volume of the activation foci and the mean signal increase elicited by stimulation of affected and unaffected eyes was calculated and subjected to a paired *t* test to assess differences between the two sides. A *P* value < 0.05 was considered significant. 

## 3. Results 

### 3.1. Functional Imaging

**Figure 2:**
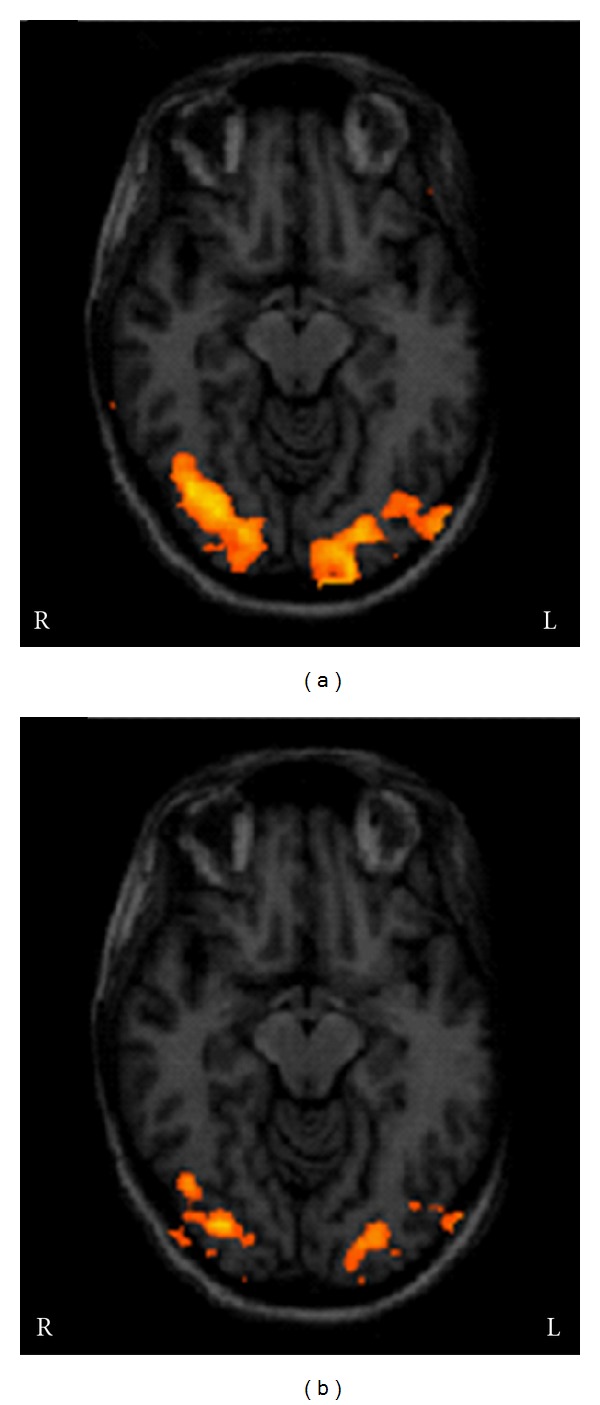
Activation foci evoked by stimulation of the unaffected (a) and affected eye (b) in a patient with acute left optic neuritis. Stimulation of the unaffected side induced bilateral activation of the visual cortex; stimulation of the affected side was associated with a reduced voxel size and BOLD signal in the activation foci. L and R: left and right.

**Figure 3:**
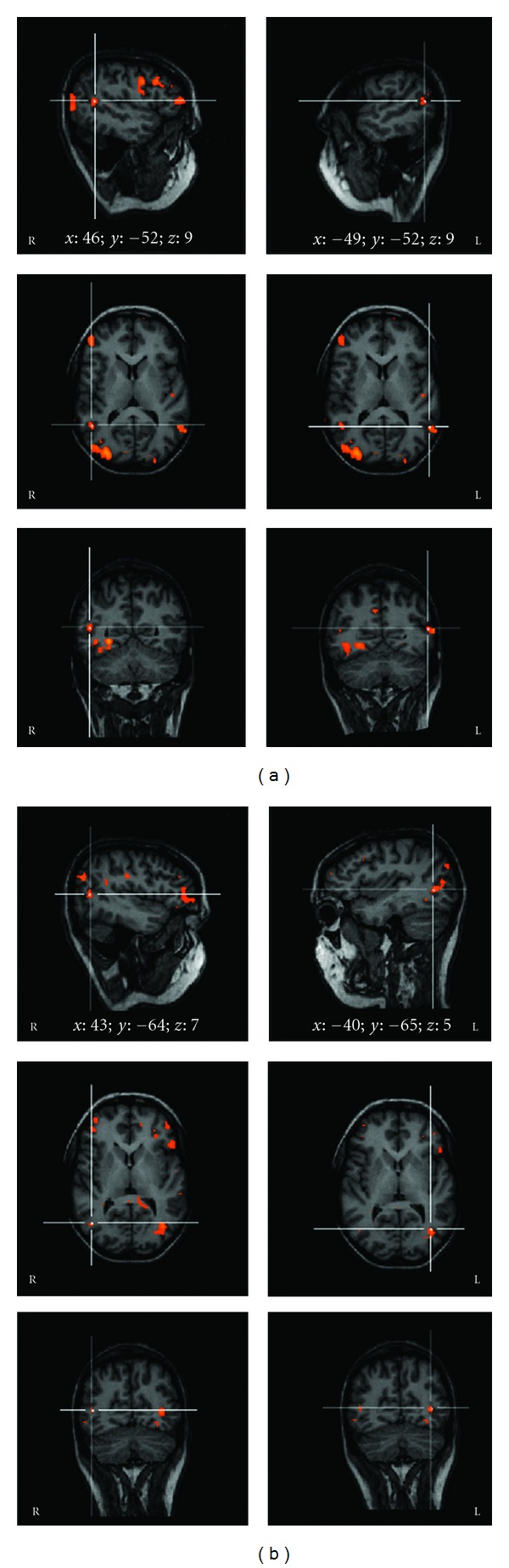
Activation foci evoked by stimulation of the unaffected (a) and affected (b) eye in a patient with acute left optic neuritis. White lines show activation in area MT/V5 in right and left hemispheres. The Talairach coordinates (*x*; *y*; *z*) of the activated area are also reported. L and R: left and right.

Visual stimulation of the unaffected eye evoked activation foci in occipitotemporal and posterior parietal areas in both hemispheres (Figure 2(a)). Blood oxygen level-dependent (BOLD) activation was also detected in frontal eye fields (FEF) and in other association areas, such as prefrontal areas and parietal eye fields. Presentation of the visual stimulus to the affected eye induced a significant reduction, and in three cases the disappearance, of activation foci in area V1 and in extrastriate areas (Figure 2(b)). Only foci in the middle temporal visual area (Figure 3(a)) and FEF were preserved. 

The volume size of the foci elicited in area V1 of both hemispheres, that is, the number of voxels activated during stimulation, ranged from 345 to 879 (mean, 576) in the unaffected eye and from 0 to 360 (mean, 70) in the affected eye; the difference was significant (*P* = 0.001). 

**Figure 4:**
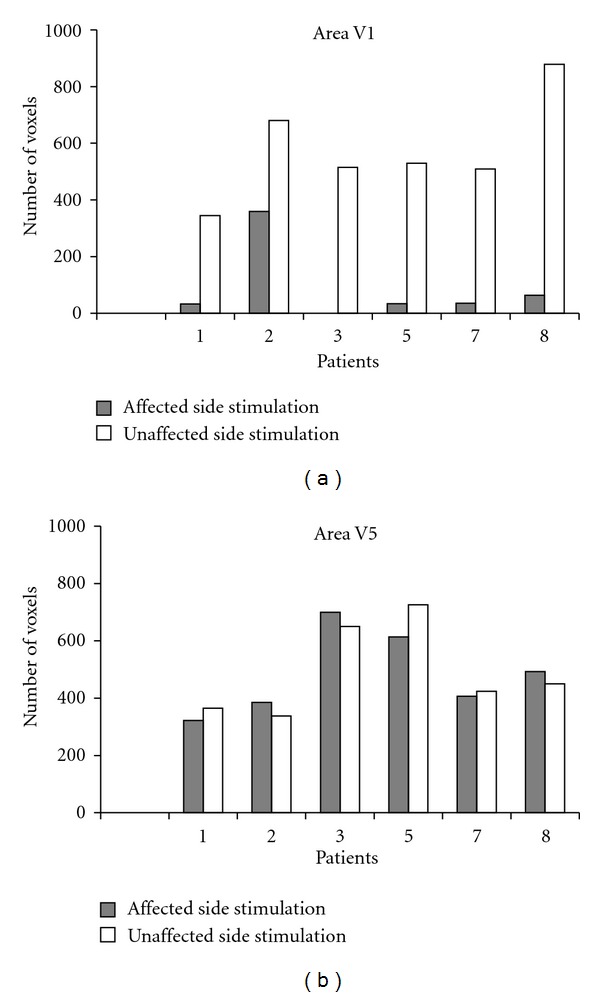
Number of activated voxels in areas V1 (a) and V5 (b) following stimulation of the affected (grey bars) and unaffected (white bars) eye in 6 patients (data of patients 4 and 6 are not provided due to bad quality of the 3D scans).

The BOLD signal increase evoked in area V1 by stimulation of the unaffected eye ranged from 0.9 to 1.7% (mean, 1.32%) and was significantly greater (*P* = 0.005) compared with the increase induced by stimulation of the affected eye, which ranged from 0 to 1.65% (mean, 0.69%), without differences between the two hemispheres (Figure 4, Table 2).

When the same measures were calculated in the foci elicited in area V5, the differences in volume size and the delay of the BOLD response evoked by visual stimulation of the unaffected and affected eye were not significant (resp., *P* = 0.4 and 0.2). In particular, the number of voxels activated during stimulation of unaffected and affected eye ranged from 338 to 726 (mean, 492) and from 322 to 700 (mean, 487), respectively. The BOLD signal increase evoked in area V5 ranged from 0.9 to 1.25% (mean, 0.96) in the unaffected eye and from 0.7 to 1.33% (mean, 0.91%) in the affected eye. (Figure 4, Table 2).

### 3.2. Diffusion Tensor Imaging

**Figure 5:**
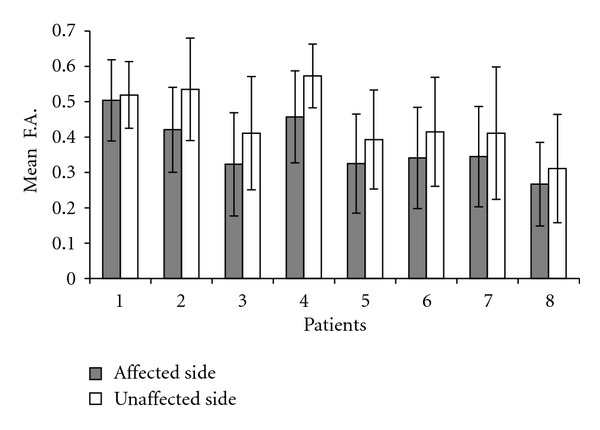
Mean FA values of optic radiations on the affected (grey bars) and unaffected side of all optic neuritis patients. Partial overlapping of error bars indicate a nonsignificant difference between affected and unaffected side anisotropy.

**Figure 6:**
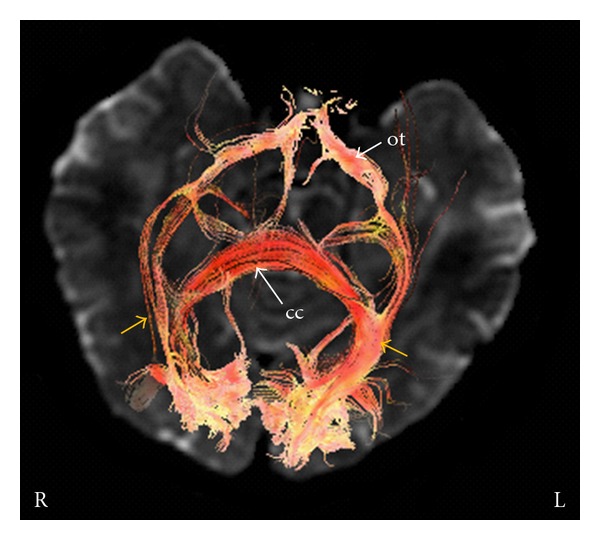
Optic radiations (yellow arrows) reconstructed using DTI fibre tracking in a patient with left optic neuritis. The two fibre bundles arise from the visual cortex and mingle with the inferior longitudinal fasciculus and inferior fronto-occipital fasciculus. Fibres also cross the splenium of the corpus callosum (cc). ot: optic tracts. L and R: left and right.

**Figure 7:**
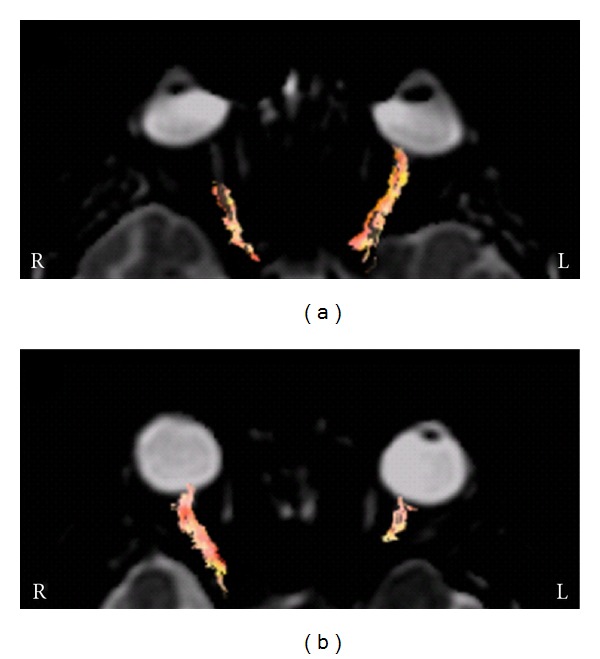
Optic nerve DTI in patients with acute right (a) and left (b) optic neuritis. DTI was able to show optic fibres in the unaffected optic nerve. At the site of the optic nerve lesion fibres cannot be reconstructed due to FA alterations; in some cases this helped define more precisely the site of the optic nerve lesion responsible for the visual impairment. L and R: left and right.

In this study DTI was applied as an adjunct to conventional MRI [15, 16]. Fibre reconstruction documented that the optic radiations were both intact and that all lesions were unilateral and involved the orbital tract of the optic nerve. The mean FA values calculated in the optic radiation of the affected side were slightly but not significantly different compared with the contralateral side (*P* > 0.05) (Figures 5 and 6). In the affected optic nerve white matter anisotropy was lower, with reduced and sometimes no visualization of streamlines in the affected tract (Figure 7). 

## 4. Discussion 

fMRI can be used to document functional damage and recovery in patients with optic nerve damage [4, 5, 17–20]. The decrease or disappearance of area V1 activation disclosed in this study can explain the vision impairment or loss experienced by ON patients and is in line with previous reports. Examination of the brain areas involved in vision during optic nerve inflammation highlighted the consistent activation of area V5 in all our patients. 

Area V5 plays an important role in motion perception and in the integration of local signals into a global perception; it is also involved in the guidance of some eye movements and projects to eye movement-related areas in the frontal (FEF) and parietal lobes (lateral intra-parietal area) [21, 22]. Visual inputs to area V5 come from areas V1, V2, and dorsal V3 [23, 24] as well as from the LGN [25] and the inferior pulvinar. The projections from LGN may explain the activation foci observed in V5 neurons even when area V1 is damaged [6–8, 25]. Some researchers who found preserved visual function despite a damaged primary visual cortex, in line with earlier physiological studies of primates, have surmised the existence of alternative visual pathways involving LGN and the middle temporal area [9, 10, 12]. Connections between LGN and area V5 have been investigated with diffusion-weighted MRI by comparing normal subjects to a blind individual [8, 11]. 

The present study describes the cortical responses elicited in patients with a damaged optic nerve but unaffected retrochiasmatic pathways and a healthy visual cortex. 

The two main pathways involved in vision [26–29], the ventral stream coursing through areas V1, V2, and V3 before reaching area V4, and the dorsal stream passing through areas V1 and V2 before reaching the dorsomedial area and area V5, are subserved by two different cell systems, the parvocellular and the magnocellular, respectively. Although they are extensively interconnected, the former system is mainly responsible for object recognition and is more sensitive to colour contrast, whereas the latter stream is mainly responsible for perception of motion and luminance contrast. Inflammatory changes are recognized as one of the major mechanisms contributing to the neural damage in multiple sclerosis and in acute optic neuritis in general [30]. Recently, a study of neuronal changes in the anterior optic pathway in multiple sclerosis patients [31] showed a greater susceptibility to injury of small parvocellular LGN neurons. 

## 5. Conclusions 

The different functional activation detected in areas V1 and V5 in our patients may be related to differences in the functional specialization and physical characteristics of the relevant pathways, the ventral and the dorsal stream. Since the dominant input to area V5 is from the magnocellular stream [32–34], the cells of this system may be preserved in ON patients, ensuring the flow of visual information along the pathway connecting LGN to area V5. 

## Figures and Tables

**Table 1:** Characteristics of the optic neuritis patients.

Patient	Age	Gender	Affected eye	Underlying pathology
	(years)			
1	70	F	Left	Idiopathic ON
2	50	F	Left	Multiple sclerosis
3	45	M	Left	Facial trauma
4	37	M	Left	Multiple sclerosis
5	23	F	Right	Idiopathic ON
6	37	M	Right	Multiple sclerosis
7	81	F	Right	Ischemia
8	28	F	Right	Multiple sclerosis

**Table 2:** Voxel number (and percent BOLD signal increase) of the activation foci elicited in areas V1 and V5 during stimulation of the affected and unaffected eye.

Patient	ON side	Area V1		Area V5	
		Left side stimulation	Right side stimulation	Left side stimulation	Right side stimulation
1	L	33 (1.65)	345 (1.7)	322 (0.7)	365 (1)
2	L	360 (1.6)	680 (1.7)	385 (1.33)	338 (1.25)
3	L	no	515 (1.5)	700 (0.85)	650 (0.9)
4^∗^	L	no	yes (—)	yes (—)	yes (—)
5	R	530 (1.5)	34 (1.5)	726 (1)	614 (1)
6^∗^	R	yes (—)	no	yes (—)	yes (—)
7	R	509 (0.9)	36 (0.6)	407 (0.95)	424 (0.9)
8	R	879 (1.3)	64 (0.6)	493 (0.67)	450 (0.7)

Data regard activation foci contralateral to the stimulation side.

^∗^For technical reason only visual localization and evaluation of activation foci was possible in patients 4 and 6.

L: left; R: right.

## References

[1] Hickman SJ, Dalton CM, Miller DH, Plant GT (2002). Management of acute optic neuritis. *The Lancet*.

[2] Jäger HR (2005). Loss of vision: imaging the visual pathways. *European Radiology*.

[3] Kupersmith MJ, Alban T, Zeiffer B, Lefton D (2002). Contrast-enhanced MRI in acute optic neuritis: relationship to visual performance. *Brain*.

[4] Korsholm K, Madsen KH, Frederiksen JL, Rowe JB, Lund TE (2008). Cortical neuroplasticity in patients recovering from acute optic neuritis. *NeuroImage*.

[5] Jenkins TM, Toosy AT, Ciccarelli O (2010). Neuroplasticity predicts outcome of optic neuritis independent of tissue damage. *Annals of Neurology*.

[6] Dancause N, Barbay S, Frost SB (2005). Extensive cortical rewiring after brain injury. *Journal of Neuroscience*.

[7] Bengtsson SL, Nagy Z, Skare S, Forsman L, Forssberg H, Ullén F (2005). Extensive piano practicing has regionally specific effects on white matter development. *Nature Neuroscience*.

[8] Bridge H, Thomas O, Jbabdi S, Cowey A (2008). Changes in connectivity after visual cortical brain damage underlie altered visual function. *Brain*.

[9] Rodman HR, Gross CG, Albright TD (1989). Afferent basis of visual response properties in area MT of the macaque. I. Effects of striate cortex removal. *Journal of Neuroscience*.

[10] Girard P, Salin PA, Bullier J (1992). Response selectivity of neurons in area MT of the macaque monkey during reversible inactivation of area V1. *Journal of Neurophysiology*.

[11] Weiskrantz L (1996). Blindsight revisited. *Current Opinion in Neurobiology*.

[12] Rosa MGP, Tweedale R, Elston GN (2000). Visual responses of neurons in the middle temporal area of new world monkeys after lesions of striate cortex. *Journal of Neuroscience*.

[13] Talairach J, Tournoux P (1988). *Co-Planar Stereotaxic Atlas of the Human Brain?*.

[14] Friston KJ, Holmes AP, Worsley KJ, Poline JP, Frith CD, Frackowiak RSJ (1994). Statistical parametric maps in functional imaging: a general linear approach. *Human Brain Mapping*.

[15] Basser PJ, Pierpaoli C (1996). Microstructural and physiological features of tissues elucidated by quantitative-diffusion-tensor MRI. *Journal of Magnetic Resonance B*.

[16] Conturo TE, Lori NF, Cull TS (1999). Tracking neuronal fiber pathways in the living human brain. *Proceedings of the National Academy of Sciences of the United States of America*.

[17] Werring DJ, Brex PA, Moseley IF (2000). Recovery from optic neuritis is associated with a change in the distribution of cerebral response to visual stimulation: a functional magnetic resonance imaging study. *Journal of Neurology Neurosurgery and Psychiatry*.

[18] Levin N, Orlov T, Dotan S, Zohary E (2006). Normal and abnormal fMRI activation patterns in the visual cortex after recovery from optic neuritis. *NeuroImage*.

[19] Korsholm K, Madsen KH, Frederiksen JL, Skimminge A, Lund TE (2007). Recovery from optic neuritis: an ROI-based analysis of LGN and visual cortical areas. *Brain*.

[20] Jenkins T, Ciccarelli O, Toosy A (2010). Dissecting structure-function interactions in acute optic neuritis to investigate neuroplasticity. *Human Brain Mapping*.

[21] Watson JDG, Myers R, Frackowiak RSJ (1993). Area V5 of the human brain: evidence from a combined study using positron emission tomography and magnetic resonance imaging. *Cerebral Cortex*.

[22] Born RT, Bradley DC (2005). Structure and function of visual area MT. *Annual Review of Neuroscience*.

[23] Ungerleider LG, Desimone R (1986). Cortical connections of visual area MT in the macaque. *Journal of Comparative Neurology*.

[24] Felleman DJ, van Essen DC (1991). Distributed hierarchical processing in the primate cerebral cortex. *Cerebral Cortex*.

[25] Sincich LC, Park KF, Wohlgemuth MJ, Horton JC (2004). Bypassing V1: a direct geniculate input to area MT. *Nature Neuroscience*.

[26] Goodale MA, Milner AD (1992). Separate visual pathways for perception and action. *Trends in Neurosciences*.

[27] Milner D, Goodale MA (1995). *The Visual Brain in Action?*.

[28] Goodale M, Milner D (2006). One brain—two visual systems. *Psychologist*.

[29] Milner AD, Goodale MA (2008). Two visual systems re-viewed. *Neuropsychologia*.

[30] Porciatti V, Sartucci F (1996). Retinal and cortical evoked responses to chromatic contrast stimuli. Specific losses in both eyes of patients with multiple sclerosis and unilateral optic neuritis. *Brain*.

[31] Evangelou N, Konz D, Esiri MM, Smith S, Palace J, Matthews PM (2001). Size-selective neuronal changes in the anterior optic pathways suggest a differential susceptibility to injury in multiple sclerosis. *Brain*.

[32] Chapman C, Hoag R, Giaschi D (2004). The effect of disrupting the human magnocellular pathway on global motion perception. *Vision Research*.

[33] Alexander KR, Rajagopalan AS, Seiple W, Zemon VM, Fishman GA (2005). Contrast response properties of magnocellular and parvocellular pathways in retinitis pigmentosa assessed by the visual evoked potential. *Investigative Ophthalmology and Visual Science*.

[34] Liu CSJ, Bryan RN, Miki A, Woo JH, Liu GT, Elliott MA (2006). Magnocellular and parvocellular visual pathways have different blood oxygen level-dependent signal time courses in human primary visual cortex. *American Journal of Neuroradiology*.

